# Prevalence of the use of homeopathy by the populationof Montes Claros, Minas Gerais, Brazil

**DOI:** 10.1590/S1516-31802009000600002

**Published:** 2010-05-21

**Authors:** João Felício Rodrigues-Neto, Maria Fernanda Santos Figueiredo, Anderson Antônio de Faria

**Affiliations:** I MD, PhD. Physician and professor in the Department of Medicine, Universidade Estadual de Montes Claros (Unimontes), Montes Claros, Minas Gerais, Brazil.; II BSc. Nurse and master’s degree student in Health Sciences, Universidade Estadual de Montes Claros (Unimontes), Montes Claros, Minas Gerais, Brazil.; III MD. Research student, Department of Medicine, Universidade Estadual de Montes Claros (Unimontes), Montes Claros, Minas Gerais, Brazil.

**Keywords:** Homeopathy, Alternative therapies, Complementary therapies, Therapeutics, Population, Homeopatia, Terapias alternativas, Terapias complementares, Terapêutica, População

## Abstract

**CONTEXT AND OBJECTIVE:**

Homeopathy is a therapeutic system that uses small doses of substances to stimulate autoregulatory and self-healing processes. The aim of this study was to investigate the prevalence of the use of homeopathy by the population of Montes Claros, Brazil, and the socioeconomic profile of users.

**DESIGN AND SETTING:**

Probabilistic cross-sectional study with cluster sampling, in the city of Montes Claros, Minas Gerais.

**METHODS:**

This study was conducted by applying semi-structured questionnaires. The sample was composed of 3,080 people. For the statistical analysis, Student’s t test and the chi-square test were used. The statistical significance level used was P < 0.05.

**RESULTS:**

We interviewed 3,090 people. The prevalence of the use of homeopathy was 2.4%. The factors associated with its use were female gender, schooling and income. The main reason that led to seeking homeopathy was “Conventional treatment did not have any effect”. For 70.2% of the users, the cost of the treatment was considered reasonable or cheap. About 73% were satisfied or very satisfied with the treatment received through homeopathy.

**CONCLUSIONS:**

The prevalence of the use of homeopathy found here was less than that reported in other countries. People with higher income and schooling levels used homeopathy more frequently. There was higher prevalence among women. Most users declared themselves satisfied with the treatment received.

## RESUMO

**CONTEXTO E OBJETIVO:**

A homeopatia é um sistema terapêutico que utiliza pequenas doses de substâncias para estimular processos de autocura e autorregulação. O objetivo deste estudo foi verificar a prevalência de utilização da homeopatia pela população de Montes Claros e o perfil socioeconômico do usuário.

**TIPO DE ESTUDO E LOCAL:**

Estudo transversal, probabilístico, por conglomerados, na cidade de Montes Claros, Minas Gerais.

**MÉTODOS:**

Estudo realizado por meio da aplicação de questionários semi-estruturados. A amostra foi composta por 3.080 pessoas. Na análise estatística, utilizou-se o teste t de Student e o teste do Qui-quadrado. A significância estatística considerada foi P < 0,05.

**RESULTADOS:**

Foram entrevistadas 3.090 pessoas. A prevalência de utilização da homeopatia foi de 2,4%. Os fatores associados à utilização foram: gênero feminino, escolaridade e renda. O principal motivo que levou a procura da homeopatia foi “o tratamento convencional não surtiu efeito”. Para 70,2% dos usuários o custo do tratamento foi considerado suportável ou barato. Cerca de 73% ficaram satisfeitos ou muito satisfeitos com o tratamento recebido na homeopatia.

**CONCLUSÕES:**

A prevalência de uso de homeopatia encontrada ficou aquém da relatada em outros países. Pessoas com melhores níveis de renda e escolaridade utilizam homeopatia com maior frequência, assim como as mulheres. A maioria dos usuários declarou-se satisfeito com o tratamento recebido.

## INTRODUCTION

Over recent years, a progressive increase in the activities of extending the use of and informing about complementary and alternative medicine (CAM) has been observed.^1^ In a functional manner, CAM can be defined as therapy that is not widely taught in medical schools or that is not usually available in hospitals.^2^ The World Health Organization (WHO) defines CAM as the general combination of types of healthcare that are not part of the tradition of a country and that are not integrated into the dominant healthcare system.^3^

The types of alternative therapies used vary from one country to another.^4^^,^^5^ The forms most commonly used and researched in international studies include: prayers to God, self-help, folk remedies, diet programs, chiropractics, physical exercise, phytotherapy, massage, acupuncture, homeopathy, Reiki and ayurveda medicine.^2^^,^^3^

CAM has attracted more and more attention from the media, the medical community, government agencies and the general public.^2^^,^^6^ Despite this increase in use, the growing interest in alternative therapies and the high costs involved, few studies have investigated the patterns of use of alternative therapies in Brazil. Among the types of CAM used in this country is homeopathy, and this forms the focus of the present article.

Homeopathy is a therapeutic system that has been in use for about 200 years. It uses small doses of substances to stimulate autoregulatory and self-healing processes.^7^ It is based on three principles: similarity, minimum doses and symptomatic totality. The first principle argues that a given substance is able to cure symptoms in a sick person when the symptoms are similar to those produced by the same substance when administered to a healthy person. In modern homeopathy, this process is called human pathogenetic testing.^7^ The second principle, which is the most controversial one, says that medicines retain biological activity when diluted in series and shaken between each dilution.^7^ Finally, there is the principle of symptomatic totality, which advocates that drugs are more effective when chosen not only because of the particular sickness**,** but also because of the individual characteristics of the patients.^7^

The effectiveness of homeopathy has been investigated in some specific clinical situations, such as in cancer treatment,^8^^,^^9^^,^^10^^,^^11^ diseases caused by *Trypanosoma cruzi*,^12^ diabetes,^13^ menstrual irregularities^14^ and other clinical conditions. Furthermore, placebo or allopathic medicine has sometimes been compared with homeopathy. However, the results have been controversial.^15^

In Europe, it has been shown that the percentages of populations using homeopathy are 28% of Danes, 31% of the Dutch, 32% of the French and 56% of Belgians.^4^ In the United States, the use of alternative medicine to care for people’s health has been systematically demonstrated.^2^^,^^6^ The expenditure on practitioners of alternative therapies increased by 45.2% between 1990 and 1997 in the United States and was estimated to be $21.2 billion in 1997.^2^ Unconventional treatments are used by many physicians and other therapists throughout Europe,^4^ Australia,^16^ China and Israel.^17^ It is estimated that the number of patients using homeopathy in the United States increased by 500% between 1990 and 1997.^2^

## OBJECTIVE

The main aims of this study were: to establish the prevalence of the use of homeopathy in Montes Claros; to discover the main reasons that lead people to seek homeopathic help and their satisfaction with the treatment received; to investigate whether the use of homeopathy has any associations with gender, schooling level, age or monthly income. Moreover, this study sought to provide a source of information for the planning of public health policies in Montes Claros and Brazil.

## METHODS

This was a cross-sectional study conducted in the city of Montes Claros, Minas Gerais. The sample was probabilistic, obtained by means of clusters within homogeneous extracts, and the sample unit was the home. The inclusion criteria were that the subjects should be over 18 years of age and be living in Montes Claros. The subjects could be of either sex. The only exclusion criterion was refusal to participate in the study.

The sample was calculated based on the expectation that the prevalence of the use of homeopathy would be 30%. This figure was obtained by reference to use in other countries, where the rate of use ranges from 3.4%^2^ to 56%,^4^ because there is no systematic population-based studies to assess the prevalence of homeopathy in Brazil. For an error of 2% and a type I error probability of 5%, it would be necessary to include 2,017 participants, according to the formula n = p * (1-p) / (e/α)^2^, where: n = sample size; p = expected proportion (30% or 0.30); e = error (2% or 0.02); and α = type I error (1.96). However, for reasons relating to the operational viability of the study, 3,080 participants were evaluated.

The sample was selected using data from the Brazilian Institute for Geography and Statistics, which divides Montes Claros into 216 census tracts. For this selection, non-urban and special urban tracts (asylums, kindergartens, orphanages, hospitals and convents), which totaled 35, were not included. This left 181 tracts, of which 163 were “urban, not special” (where about 96% of the urban population lives) and 18 were “urban subnormal”, i.e. slums or shantytowns (around 4% of the urban population).

In the second stage, a draw was carried out to determine the order of households to be visited within a tract. The cluster size chosen was three households. The households were thus divided into threes, and the first out of each three was visited. Since each census tract had an average of 200 to 300 homes (and one out of three would be visited), 35 sectors were put into the draw. Of these, 31 were “urban, not special” (where 96% of the interviews took place) and four were “urban, subnormal” (where 4% of interviews took place). Through this procedure, the sample size that had been calculated was reached.

The data collection instruments used were 3,090 semi-structured questionnaires containing 49 questions each. These were applied by five graduates in health-related subjects who had been given specific training for the task. Over an initial two-week period, pilot interviews were conducted and the questionnaires were subsequently reviewed.

The interviews for the main study were then conducted. If it was not possible to conduct an interview, either because the home did not exist or there was nobody at home, the next home was visited and counting was resumed from there. The length of time for which an individual had lived in the census tract was not considered to be a criterion for inclusion or exclusion.

The questionnaires were gathered in every week and checked by the field coordinator. If there was a failure to fill in forms or to collect the data, the interviewer was notified and that particular questionnaire was excluded.

The variables studied were the different forms of alternative therapy that were in use. Thus, the therapies investigated included acupuncture, homeopathy, massage, diet programs, orthomolecular medicine, folk remedies, chiropractics, physical exercises, spiritual healing by others, relaxation and meditation techniques, self-help groups, praying to God, spiritual guides, gurus and spiritist centers.

To assess the interviewees’ socioeconomic characteristics, the monthly family income and schooling level that they declared were used. The level of physical health was investigated using 1) the interviewees’ own self-reports, on a scale from one (“excellent”) to five (“bad”), and 2) the presence of health problems, noting that up to six comorbidities could be recorded for each interviewee.

The following demographic characteristics were studied: gender, age, skin color, marital status, economic activity, schooling, number of people living together and monthly family income.

For the statistical analysis, the data were recorded and analyzed using the Statistical Package for the Social Sciences (SPSS) software, version 15.0 for Windows. Descriptive statistics were used to record the data. Student’s t test was used for comparisons between means, and the chi-square test was used for comparisons between proportions. The statistical significance level used was P < 0.05.

This study was conducted within the standards required by the Declaration of Helsinki and was approved by the Research Ethics Committee of Universidade Estadual de Montes Claros (Unimontes). All of the participants signed a free and informed consent statement, after agreeing to take part in the study.

## RESULTS

Interviews were conducted with 3,090 people, and the use of homeopathy was identified in 74 cases, i.e. a prevalence of 2.4%. The main reasons that led people to seek homeopathic treatment were that conventional treatment had no effect (16; 21.6%); they were seeking something better (13; 17.6%); a physician had recommended it (8; 10.8%); it would complement conventional treatment (7; 9.5%); and conventional treatment was very expensive (2; 2.7%). Twenty-eight interviewees (37.8%) were unable to give an answer.

The cost of the homeopathic treatment for the patient or the family was reported to be affordable for 34 (45.9%); cheap for 18 (24.3%); and expensive for 14 (18.9%). Eight interviewees (10.9%) were unable to give an answer. Regarding satisfaction, 54 (73%) reported being satisfied or very satisfied with the homeopathic treatment, while 15 (20.3%) were somewhat satisfied or dissatisfied with the treatment received. Five (6.7%) made no comment.

In the full sample, 1,936 (62.65%) were female; and among the users of homeopathy, females numbered 62 (83.8%) (P = 0.00) (Table 1). The mean age ± standard deviation among non-users was 39.61 ± 16.26 years; among users, it was 41.38 ± 15.14 years. The predominant skin color among non-users of homeopathy and among users was white, followed by brown/mixed (P = 0.805) (Table 1). A monthly family income of less than two minimum salaries was common in the full sample and income of more than two minimum salaries predominated among the users (P = 0.006) (Table 1).

Higher schooling levels were recorded as common among the people who used homeopathy. In percentage terms, the number of people who had completed more advanced schooling levels was five times higher among the homeopathy users. Among the non-users of homeopathy, the most common schooling level was incomplete elementary education, while among users of homeopathy the most common level was completed high school (Table 1).

In terms of professional activities among the homeopathy users, 37 (50%) were without work at the time of the interview, 35 (47.3%) worked in the service sector and two (2.7%) in industry. None of the users reported working in the agricultural sector.

Among those not using homeopathy, 691 (23.0%) reported having had depression at some time, while among the users, this number was 23 (35.1%). Similarly, among the non-users, 1,019 (33.8%) had a health problem, while among the users, the number was 36 (48.6%). The analysis on the interviewees’ self-reported health showed that among the users of homeopathy, 26 (35.1%) considered their health to be fair, 22 (29.7%) good, 13 (17.6%) very good, eight (10.8%) excellent and five (6.8%) bad. The main health problems found in this group were: hypertension (seven individuals, 9.4%), allergy (four individuals, 5.4%) and bronchitis (four individuals, 5.4%), followed by depression, sinusitis and hypercholesterolemia. These were not necessarily the reason that prompted them to seek homeopathic help.

Both the users and the non-users of homeopathy considered it reasonable that the public health system should make homeopathy available as a therapeutic option (Table 1).

**Table 1.** Cultural and sociodemographic profile of the 3,090 interviewees

Characteristic	Does not use homeopathy		Uses homeopathy		Value
	n	%	n	%	P
Gender					
Male	1142	37.9	12	16.2	0.000
Female	1874	62.1	62	83.8	
Age (years)					
18-30	1080	35.8	23	31.1	0.128
31-50	1148	38.1	27	36.5	
51-60	375	12.4	16	21.6	
Over 60	413	13.7	8	10.8	
Color					
Brown/mixed	1314	43.6	29	39.2	0.805
White	1339	44.5	37	50.0	
Black	311	10.3	6	8.1	
Oriental	47	1.6	6	2.7	
Other	1	0.0	0	0.0	
Marital status					
Married or lives with partner	1576	52.3	41	55.4	0.604
Single or widowed or separated	1440	47.7	33	44.6	
Schooling					
Illiterate	164	5.4	2	2.7	0.000
Elementary education incomplete	1011	33.6	13	17.6	
Elementary education complete	392	13.0	3	4.1	
High school complete	217	7.2	2	2.7	
High school incomplete	964	32.0	29	39.2	
University-level complete	111	3.7	6	8.1	
University-level incomplete	151	5.0	19	15.7	
Family income in monthly minimum salaries					
Up to 1 minimum salary	521	17.3	6	8.1	0.000
1 to 2 minimum salaries	1093	36.2	18	24.3	
2 to 4 minimum salaries	939	31.1	17	22.9	
4 to 8 minimum salaries	354	11.8	21	28.4	
8 to 16 minimum salaries	91	3.0	8	10.8	
Over 16 minimum salaries	18	0.6	4	5.4	
Do you consider it reasonable for the health system to offer homeopathy as a therapeutic option?					
Yes	72	97.2	2037	67.5	0.000
No	1	1.4	78	2.6	
Indifferent	0	0.0	39	1.3	
Don’t know	1	1.4	862	28.6	

## DISCUSSION

The results from this population-based study were obtained from a representative sample of the population of adults (both men and women) in a midsize city in southeastern Brazil.

The data on the use of homeopathy in Brazil is still rather sparse. The prevalence of the use of homeopathy found in this study was lower than that shown in other countries, such as the United States^2^ and some European countries.^4^ Most of these other studies included pediatric and adolescent age groups. This may partially explain the differences found, since the present study only included people over the age of 18, whereas homeopathy is an alternative therapy that is widely used in the pediatric age range. Specific use with this age group has been shown in Canada, England and Italy.^18^

Socioeconomic factors may also have influenced the results, since the costs involved limit the use of homeopathy in Brazil. This is because a specialist in this field is necessary, but the public health system does not cover this yet. Thus, homeopathy is largely restricted to a small portion of the population that has the means to afford the treatment.

The higher prevalence of use among females in this study was similar to that found by Jacobs et al.^19^ for homeopathy and by Eisemberg et al.,^2^ Zollman and Vickers,^20^ Lim et al.^21^ and Hanssen et al.^3^ for alternative therapies in general.

Although the age groups considered in the various studies were not identical, they were similar. The age range over which there is evidence of highest demand coincides with the period of greatest economic productivity, which may show that there was less dependence on public services and more autonomy in choosing healthcare methods. In this present study, neither age nor skin color was associated with choosing homeopathic methods. In the United States, blacks use less homeopathy than do whites.^19^ In Brazil, the reality of extensive racial mixing may have influenced the analysis on this variable.

Comparison of monthly family incomes between users and non-users of homeopathy showed that users had higher income levels as well as higher schooling levels. In our results and in another study conducted in Santos, Brazil,^22^ high income and schooling levels were associated with increased use of homeopathy. The same was demonstrated among users of homeopathy in the United States^19^ and among users of alternative therapies in general in other countries,^2^^,^^20^^,^^23^ but especially in relation to those therapies involving expenditure. This can be explained by the need to pay the expenses incurred in appointments with physicians and in treatment. While greater levels of information open up the range of treatment options, the availability of resources promotes the costs involved.

Most of the conditions that homeopathy treats are chronic or recurrent.^24^ In the United States, out of the ten most common diagnoses among people who sought homeopathic physicians, nine involve chronic diseases.^19^ The main diseases reported by users of homeopathy in the present study were also chronic or recurrent: hypertension, allergies, bronchitis and depression. They are not necessarily the reason for seeking homeopathy.

States of emotional morbidity, assessed through reports of depression, have shown links between such states and seeking homeopathic treatment. Depression is the second most common diagnosis among patients seeking homeopathic physicians in the United States,^19^ while the most common is asthma. In Latin American societies, the search for new therapeutic options is in line with the limitations of allopathic medicine, such as its limited effectiveness for treating diseases with a strong psychological component.^24^

An experimental study found that homeopathic medication was able to significantly reduce anxiety in the population studied, compared with placebo in surgical procedures.^25^

The increased prevalence of chronic diseases and access to information about health, as well as the increasing popularity of alternative therapies, reflects changes in the values of modern societies in general.^17^ In our study, a significant number (37.8%) of the interviewees were unable to give a reason why people seek homeopathic help, perhaps because this is seen as a new option for treating their needs. Among those who could give a reason for their use of homeopathy, lack of improvement (“did not help”) through using conventional medicine was the main justification for seeking homeopathy that was shown by the present study, followed by “searching for something better”.

A Brazilian study^22^ found that the main reason for the demand for homeopathic treatment was dissatisfaction with allopathic treatment, and this result was also found in the present study. The lack of effectiveness of conventional therapy was also put forward by Pal^17^ to explain the demand for alternative therapies. The main factors that this author quoted were: 1) Dissatisfaction with conventional therapy, because of ineffectiveness, adverse effects or its increasingly technological nature; 2) The need for personal control: patients believe that in choosing alternative therapies, they have more control over their healthcare; and 3) Alternative therapies are seen as more compatible with patients’ values, their view of the world and their spirituality and religiosity.

Research among users of complementary medicine has indicated that about 80% are satisfied with the treatment received.^17^ The satisfaction with the treatment received through homeopathy was found, in the present study, to be different from the satisfaction with conventional treatment. The satisfaction with conventional treatment was lower (62.94% were satisfied or very satisfied). However, it should be taken into account that no distinction was made between the treatments provided by the private and public health systems when satisfaction with conventional therapies was evaluated. The limitations inherent to public health services in Brazil, such as the time spent in waiting, shorter time available for consulting with physicians and infrastructure problems, undoubtedly contributed towards reducing the satisfaction level. On the other hand, the homeopathic consultations were all private, and therefore less influenced by the factors cited above.

Most of the individuals who use homeopathy considered that the expenditure involved was affordable or cheap, although the family income levels among users were higher than those of non-users. This has particular relevance in view of the lack of homeopathic physicians in the public health service, which means that it is users or their families who pay the expenses. It has been suggested that the costs involved in health care can be reduced by using such therapy.^19^ A study in France showed that the per capita costs incurred through homeopathy may be 15% lower than with conventional doctors. This saving was attributed to the lower need for diagnostic tests and also the lower cost of homeopathic medicines. No research analyzing whether insurance companies in Brazil cover homeopathy was found. In other countries, a growing number of insurers are offering coverage for alternative therapies.^19^

The costs involved in alternative therapies may be lower and their effectiveness in helping treat certain diseases may be greater.^19^ In excluding therapeutic alternatives to allopathic medicine, health systems are not maintaining their goal of meeting the needs of all the population.^24^ The population surveyed considered that inclusion of homeopathy in the public health system was reasonable. When only the opinions of those who use homeopathy were examined, this number was considerably higher, thereby reinforcing the need for provision of homeopathy within the Brazilian National Health System (SUS). Thus, for homeopathy, the National Policy for Integrative and Complementary Practice that was approved by the Ministry of Health in May 2006 needs to be implemented,^26^ so that its use will not be restricted to the economically advantaged classes.

In developing countries, Integrative and Complementary Practice (ICP) is called Traditional Medicine (TM), whereas it is called Complementary and Alternative Medicine (CAM) in developed countries.^27^ In the present study, although homeopathy is a medical specialty and cannot be called alternative medicine, the nomenclature Complementary and Alternative Medicine was used to adapt to the international designation used by developed countries.

The potential limitations of the results from the present study might include reverse causality, which is a frequent occurrence in interpreting the results from cross-sectional studies. However, the results from this study were interpreted with great caution, precisely because of the possibility of bias.

Further studies are needed, preferably multicenter in nature, in order to obtain information that could promote health practices aimed at inclusion of the use of complementary medicine in most Brazilian municipalities, ranging from the field of vocational training to the provision of these therapies for healthcare.

## CONCLUSION

Homeopathy is not widely used in Montes Claros, but the majority of the users reported being satisfied with the treatment received. The predominant groups among these users were women, people with higher income levels and people with higher schooling levels. Among the reasons for the demand for homeopathy that are known and reported, the main one related to lack of results from conventional medicine. Most respondents, regardless of whether or not they used homeopathy, considered it reasonable for the public health system to make homeopathy available to the population.
